# Occupational cancer illness in Brazil: an integrative literature review

**DOI:** 10.47626/1679-4435-2022-845

**Published:** 2023-08-08

**Authors:** Thamyres Morgado de Almeida, Yasmin Albuquerque da Costa, Magda Guimarães de Araujo Faria, Cristiane Helena Gallasch

**Affiliations:** 1 Faculdade de Enfermagem, Universidade do Estado do Rio de Janeiro, Rio de Janeiro, RJ, Brazil

**Keywords:** occupational health, occupational risks, occupational cancer, saúde do trabalhador, riscos ocupacionais, câncer ocupacional

## Abstract

The present study aimed to investigate data from the scientific literature on patterns of illness due to occupational cancer in Brazil. An integrative literature review was conducted in July 2020 and reviewed in June 2021, with no time restriction, using the Health Science Descriptors “Neoplasms,” “Occupational Risks,” “Occupational Cancer,” and keywords related, which were searched on the following databases: Latin American Literature in Health Sciences, SciELO, PubMed, Scopus, Web of Science, and Cumulative Index to Nursing and Allied Health Literature. The search and selection flow followed that recommended by the Preferred Reporting Items for Systematic Reviews and Meta-Analyses Statement – 2020. Six manuscripts were selected, published from 1995 to 2019, which described occupational cancer affecting lung, oral cavity, pharynx and larynx, central nervous system, and skin. There was a time gap between the studies, and only the one involving lung cancer results on the most prevalent pattern of illness in the industrial sector, compared to the non-industrial. A shortage of scientific articles on patterns of illness was found. There was a predominance of scientific publications referring to occupational cancer illness related to the industrial sector compared to the number of publications directed to the primary and tertiary productive sectors. It is worth noting the constant need for research and epidemiological surveys to promote surveillance and protective actions aimed at occupational health.

## Abstract (pt)

O presente estudo teve como objetivo investigar os dados da literatura científica sobre os padrões de adoecimento por câncer ocupacional no Brasil. Realizou-se uma revisão integrativa de literatura, em julho de 2020 e revisada em junho de 2021, sem delimitação temporal, utilizando os Descritores em Ciências da Saúde “Neoplasias”, “Riscos Ocupacionais” e “Câncer Ocupacional”, e correlatos, com busca nas bases Literatura Latino-Americana em Ciências da Saúde, SciELO, PubMed, Scopus, Web of Science e Cumulative Index to Nursing and Allied Health Literature. O fluxo de busca e seleção obedeceu ao preconizado pelo Principais Itens para Relatar Revisões Sistemáticas e Meta-Análises – 2020. Foram selecionados seis manuscritos, publicados entre 1995 e 2019, que descrevem o câncer ocupacional relacionado a pulmão, cavidade oral, faringe e laringe, sistema nervoso central e pele. Há um hiato temporal entre os estudos e apenas aquele envolvendo câncer de pulmão considera resultados sobre o padrão de adoecimento mais prevalente no setor industrial, quando comparado ao não-industrial. Foi constatada a uma escassez de artigos científicos sobre padrões de adoecimento. Há predomínio de publicações científicas referentes ao adoecimento por câncer ocupacional relacionado ao ramo industrial quando comparado ao quantitativo de publicações direcionadas aos setores produtivos primário e terciário. Ressalta-se que pesquisas e inquéritos epidemiológicos são necessidades constantes para promoção de ações vigilância e proteção da saúde no trabalho.

## INTRODUCTION

Occupational health is a field of public health that gathers a body of knowledge on different areas, such as social medicine, public health, clinical medicine, occupational medicine, and occupational nursing, as well as law, sociology, social epidemiology, engineering, and psychology. In this context, its purpose is to identify and intervene in work and health-disease relationships; furthermore, understanding the relationship between work and disease is a paradigm for systematizing occupational health practices.^1,2,3^

The workers’ morbidity and mortality profile in Brazil is defined by the coexistence of injuries related to specific working conditions, such as occupational accidents and professional diseases, which are named work-related diseases.^4^ There are several causes for illness among workers, and neoplasms are increasingly more present both in the general population and in the organizational sphere.

Work-related cancer originates from exposure to carcinogenic agents present in the work environment, even after ceasing exposure.^5^ In Brazil, the Ministry of Health, through its specialized technical departments, has sought to estimate cancer-related occupational and environmental factors and intervening in them, formulating health surveillance procedures. An important landmark was the development of guidelines on work-related cancer, which subsidize the Brazilian Unified Health System, especially the Brazilian Network of Comprehensive Occupational Health Care.^6^

Therefore, the main strategy to reduce occupational risks for cancer is reducing or eliminating exposure to agents classified as carcinogenic. However, it is worth considering the dynamic process between occupational exposure and cancer, taking into account the frequent changes in the characteristics of different occupations, or even the disappearance of some of them, which give way to the emergence of others, in addition to the large number of substances produced in industrial processes.^7^

Considering the relevance of the theme and the need for information on the occurrence of occupational cancer among Brazilian workers, it is wondered which data are available on scientific literature on patterns of cancer illness associated with occupational exposure in Brazil. The present study aimed to investigate data from the scientific literature on patterns of occupational cancer illness in Brazil.

## METHODS

This is an integrative literature review, a research method that considers a group of scientific productions on a given subject, aiming to systematize and synthesize data from already published studies.^8^ The six steps recommended for this method were followed, starting with the definition of the topic and elaboration of the guiding question through the PICO strategy, defining population (P) as workers, phenomenon of interest (I) as patterns of cancer illness, and context (CO) as occupational cancer. Therefore, the following research question was formulated: What data are presented in the scientific literature on patterns of cancer illness associated with occupational exposure in Brazil?

The second stage established inclusion and exclusion criteria for the studies. Inclusion criteria consisted of publications related to the Brazilian working population, with full text available, and published in Portuguese, English, or Spanish, with no time restriction. Duplicate articles were excluded, as well as theoretical and reflexive manuscripts, literature reviews, and studies that did not address the research question.

The search was conducted using the following Health Science Descriptors: Neoplasms, Occupational Risks, and Occupational Cancer, and the Boolean operators [AND] and [OR]. Furthermore, it was carried out in July 2020, and subsequently reviewed by two researchers in June 2021, on the Latin American and Caribbean Health Sciences Literature (Literatura Latino-Americana em Ciências da Saúde), SciELO, PubMed, Scopus, Web of Science, and Cumulative Index to Nursing and Allied Health Literature databases. Chart 1 presents the syntaxes applied to these databases.

**Chart 1:** Syntax between descriptors and Boolean operators applied in searches on databases, Rio de Janeiro, RJ, Brazil, 2021

LILACS: ((“Riscos ocupacionais” OR “Condições Inseguras no Trabalho” OR “Risco Ocupacional” OR “Risco Profissional” OR “Trabalho Precário” OR “Riesgos Laborales” OR “Condiciones Inseguras en el Trabajo” OR “Riesgo Laboral” OR “Riesgo Profesional” OR “Riesgos Profesionales” OR “Occupational Risks” OR “Insecure Labor Conditions” OR “Occupational Risk” OR “Work Risk”) AND (“Brasil” OR Brazil) AND (“Neoplasias” OR Câncer OR Neoplasia OR “Neoplasia Benigna” OR “Neoplasia Maligna” OR “Neoplasias Malignas” OR Neoplasmas OR Tumor OR “Tumor Maligno” OR Tumores OR “Tumores Malignos” OR Neoplasms OR “Benign Neoplasm” OR “Benign Neoplasms” OR Cancer OR Cancers OR Malignancies OR Malignancy OR “Malignant Neoplasm” OR “Malignant Neoplasms” OR Neoplasia OR Neoplasias OR Neoplasm OR Tumor OR Tumors) OR (“Câncer Ocupacional”) OR “Câncer Ocupacional” OR “Cáncer Profesional” OR “Cáncer Laboral” OR “Occupational Cancer”))
SciELO: ((Occupational Risks AND (Neoplasms OR Occupational Cancer)); (neoplasias AND riscos ocupacionais AND (câncer ocupacional); ((Câncer Ocupacional OR Neoplasias) AND Adoecimento)
PubMed: ((“Neoplasms” OR “Occupational Cancer”) AND “Occupational Risks”)
Scopus: ((“Neoplasms” OR “Occupational Cancer”) AND “Occupational Risks”)
Web of Science: ((“Occupational Risks” AND (Neoplasms OR “Occupational Cancer”))
CINAHL: ((neoplasms OR “Occupational Cancer”) AND “Occupational Risks”)

LILACS = Latin American and Caribbean Health Sciences Literature (Literatura Latino-Americana em Ciências da Saúde); CINAHL = Cumulative Index to Nursing and Allied Health Literature; SciELO = Scientific Electronic Library Online.

**Figure 1:**
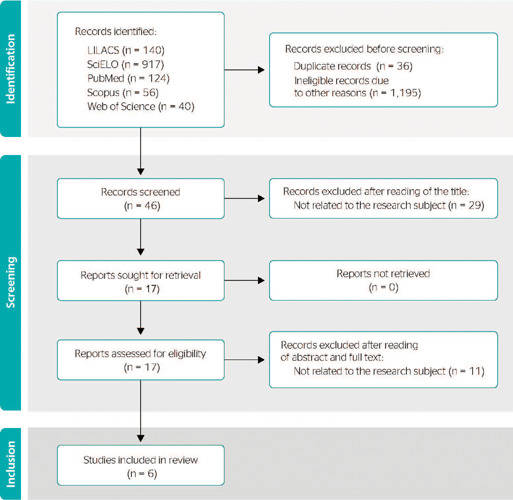
Information resources consulted, search strategies, references retrieved and selected, Rio de Janeiro, RJ, Brazil, 2021. LILACS = Latin American and Caribbean Health Sciences Literature (Literatura Latino-Americana em Ciências da Saúde); SciELO = Scientific Electronic Library Online.

In the third stage, related to the assessment of study eligibility, we used the Preferred Reporting Items for Systematic Reviews and Meta-Analyses (PRISMA 2020) flowchart, presented in four stages: 1. Identification – number of texts found according to database and number of studies remaining after exclusion of duplicate ones; 2. Selection – number of selected and excluded publications; 3. Eligibility – analysis of full texts, describing those included in and excluded from the qualitative synthesis; and 4. Inclusion – total number of studies included in the qualitative synthesis.^9^ Figure 1 shows the flowchart describing the eligibility and inclusion of articles in the studies selection.^9^

In the fifth stage, we aimed to interpret the results of the studies selected and included in the final sample. In order to outline the analyzed information retrieved from the studies selected in the third stage, a table was organized with data from the collected articles containing the following information: title of the article, country of publication, year of publication, objective of the study, database, and main results.^8^ The sixth stage, presented in the Results session, describes the synthesis of knowledge on the subject of this study.

## RESULTS

Initially, it was found that the topic addressed in this review has not been previously published. Based on inclusion and exclusion criteria, six manuscripts were selected. The synthesis of the findings is presented in Chart 2.

**Chart 2:** References selected for quantitative synthesis, Rio de Janeiro, RJ, Brazil, 2021

Year/study design	Substances, circumstances of exposure or occupations	Main results
1995^10^/case-control study	Industrial work Lung cancer	Workers linked to the production sectors of several industries have twice the risk of developing lung cancer compared to workers involved in non-industrial activities.
2000^11^/observational exploratory study	Industrial work Central nervous system cancer	A statistically significant high mortality was observed in the industrialized area, for ages over 10 years in all periods, and only from 1980-1993 for ages from 34 to 64. This was probably related to the occupational and environmental exposure to carcinogens found in the in the port and industrial complex.
2006^12^/case-control study	Activities in vehicle maintenance shops Oral cavity and oropharyngeal cancer	Working as vehicle mechanic represents a risk situation for oral cavity and oropharyngeal cancer, regardless of age, smoking, and alcohol. More prolonged exposure increases the risks.
2007^13^/case-control study	Laryngeal cancer	Associations between laryngeal cancer and occupational exposure to respirable free crystalline silica, soot (from coal, coke, wood, or fuel oil), fumes, and live animals are not explained by smoking patterns or alcohol consumption.
2015^14^/observational-exploratory study	Rural workers exposed to ultraviolet radiation and pesticides Skin cancer	97.7% of rural workers participating in the study are exposed to physical and chemical risks – ultraviolet radiation and pesticides –, 5.4% were identified with a previous diagnosis of skin cancer.
2019^15^/cross-sectional study	Oral cavity and oropharyngeal cancer	Workers in occupations related to trade, construction and cleaning, household, hotel and building maintenance represented for the largest number of cases of oral cavity and oropharyngeal cancer. However, the distribution of cases differed when they were analyzed according to the presence of smoking and alcohol consumption.

The sample of this review consisted of studies published from 1995 to 2019 and that addressed the risk for development of lung cancer,^10^ central nervous system cancer,^11^ oral cavity, pharyngeal, laryngeal, and skin cancer related to exposure to pesticides and solar radiation.^12,15^ It was also observed that the compiled studies described investigations on the primary sector, with rural work,^14^ secondary sector, including industry,^10,11^ and tertiary sector, including trade, construction, and cleaning.^15^ In the 1990s, a study highlighted that industry workers have twice the risk of developing lung cancer.^10^

## DISCUSSION

Cancer is a disease that requires a long time for the body to react to the previous stimulus. Therefore, it is difficult to determine what carcinogenic agent is related to the development of this disease, since there may a specific carcinogenic agent for each type of cancer.^16^ On average, 20% of all cancers are associated with risk factors present in the workplace; however, there is a shortage of studies indicating an association between occupational risk factors with some types of cancer.^16,17^ It is worth noting that nearly 40% of all types of cancer are preventable, including those related to occupational exposure.^5^ Although occupational cancer is a relevant theme to researchers, in addition to deserving attention from employers, due to its preventable characteristics and its potential for early mortality and years of work lost,^5^ the present study highlights the small number of scientific articles found, which were published in a considerable time gap and in which the large Brazilian working population are epidemiologically explored.

The work environment where an individual is inserted may present several types of carcinogenic agents. It is worth noting that the process of cancer development is multicausal, since uncontrolled cell multiplication involves genetic, hereditary, and environmental factors, as well as dietary habits, smoking, and exposure to radiation, pesticides, and toxic chemical agents in the workplace. Furthermore, industries and companies are constantly held responsible for not protecting workers from toxic exposures and for not providing treatment to workers who were somehow affected by exposures.^17,18^

Despite the relevance of the fact that occupational factors may cause or even increase the risk of cancer in workers, the percentages found in Brazil are considered lower than estimates from other countries, since the risk attributable to occupational cancer is only 2.3% in men and 0.3% in women.^19^ However, this is considered a relevant topic, since cancer is a preventable disease.

Among the types of cancer found in publications, it is worth mentioning that lung cancer is the second most frequent in Brazil and the one with the highest incidence and mortality worldwide. According to the Brazilian National Cancer Institute, 85% of the cases are caused by tobacco use.^20^ However, individuals who work in construction, agribusiness, and especially those who handle minerals, are twice more prone to develop lung cancer than non-industrial workers.^10,18,21,22^

Asbestos is the most known and the most frequent cause of lung cancer. Nonetheless, several other occupational exposures are also proven risk factors, including exposure to crystalline silica, exhaustion gases such as diesel, polycyclic aromatic hydrocarbon, several metals (arsenic, cadmium, beryllium, certain components of chromium and nickel), soldering vapor, and ionizing radiation. However, there is still uncertainty about other probable occupational lung carcinogens, such as bitumen or arsenic-free insecticides.^18,20,22^

Moreover, in relation to the types of cancer cited in the studies, those affecting the oral cavity and the pharynx have a low incidence in the population, with average mortality rates of 1.87/100,000 and 2.04/100,000, respectively. Both types of cancer are related to smoking, alcohol consumption, and human papillomavirus in the general population.^23^

Oral cavity cancer is a type of malignancy that spreads silently, with initial lesions beings found predominantly in the format of shallow ulcers with elevated borders usually confounded with aphthae. Studies show that formaldehyde, phenoxy herbicides, and dioxins are the most found in cases of occupational oral cavity cancer, due to exposure the already described risk factors. In this study, such diagnostic was associated with employment in vehicle maintenance shops.^12^

Additionally, a relationship has already been described between nasopharyngeal cancer and rubber and aluminum industries and activities linked to painting, agriculture, and mining, with exposure to asbestos, benzene, diesel, formaldehyde, nickel, leather dust, wood dust, coal soot, organic solvents, solar radiation, pesticides, and silica.^19,24^ With regard to the relationship between occupational cancer and productive sector, it was found that exposure to asbestos, formaldehyde, silica, coal soot, organic solvents, pesticides, among others, may lead to the development of laryngeal cancer, which mainly affects farmers, miners, hairdressers, and painters.^25^

Central nervous system cancer, in turn, is seen as multifactorial, since it originates from an array of genetic and hereditary changes and occupational exposure to arsenic, lead, mercury, mineral oil, and radiation (X and gamma rays). The last-mentioned factor can affect health care professionals who perform procedures related to radiographs and radiofrequency therapies on a daily basis. Furthermore, individuals working at refineries, nuclear plants, companies involved in the production and repair of motor vehicles, as well as in the petrochemical, rubber, plastic, graphic, paper, textile, and pesticide industries, are susceptible to the mentioned carcinogenic agents.^25,26^

Finally, skin cancer was mentioned in a study involving exposure of rural workers to solar radiation and pesticides.^14^ In addition to these workers, gardeners, construction workers, as well as those working in agriculture, farming, and fishing, are also exposed to ultraviolet solar radiation, being more prone to develop non-melanoma skin cancer, as well as workers from the agricultural sector.^27^

## CONCLUSIONS

A shortage of scientific articles on pattern of occupational cancer illness was found. The *Atlas do Câncer Relacionado ao Trabalho no Brasil,* by Fundação Oswaldo Cruz (Fiocruz) stands out as the source with the greatest potential to provide data on the topic.

With regard to the occurrence of the aforementioned disease, only one study, conducted in the late 1990s, mentioned that industry workers have a two-fold higher risk of developing lung cancer, with special emphasis on the presence of numerous carcinogenic substances to which they are daily exposed. However, since then productive and economic processes underwent several restructurings, which lead to the need for monitoring the changes in these patterns of illness.

Reducing or eliminating exposure to agents classified as carcinogenic in the workplace stood out as the main strategy to reduce occupational risks for cancer, considering the dynamic relationship between exposure and illness. Furthermore, the constant need for research and epidemiological surveys to promote surveillance and protection actions aimed at occupational health should be emphasized. Thus, it is possible to recognize that organizational changes are required to reduce the rates of occupational cancer by means of redirecting models of work organization and management.
